# Bringing usability testing to the front lines: IV smart pump evaluation with critical care nurses at a national conference

**DOI:** 10.1016/j.mex.2026.104140

**Published:** 2026-09-01

**Authors:** Brenda Abena Nyarko, Seonhun Lee, Gina L. Georgadarellis, Frank C. Sup IV, Karen K. Giuliano

**Affiliations:** a Elaine Marieb Center for Nursing and Engineering Innovation, University of Massachusetts Amherst, MA, USA; b Elaine Marieb College of Nursing, University of Massachusetts Amherst, MA, USA; c Riccio College of Engineering, University of Massachusetts Amherst, MA, USA; d Institute for Applied Life Sciences, University of Massachusetts Amherst, MA, USA; e Baystate Health, Springfield, MA, USA

**Keywords:** Human factors, Usability testing, Eye tracking, Subjective workload, Nurse-device interaction, Infusion technology, IV smart pumps, Conference-based recruitment

## Abstract

Intravenous smart pump (IVSP) usability evaluations are often conducted in single-site laboratory or simulation settings, which can limit access to experienced frontline nurses. This methods article describes a field-deployable, conference-based approach for evaluating IVSP usability with practicing critical-care nurses. The method brought a standardized simulation environment to a national critical-care nursing conference while preserving experimental control through consistent pump stations, scripted clinical scenarios, counterbalanced device order, and common data-collection procedures. The approach integrates direct observation of programming performance with wearable eye tracking, physiologic monitoring, subjective workload ratings, healthcare-specific usability assessment, think-aloud comments, and device preference ranking. The method is intended for researchers, health systems, manufacturers, and nurse-engineer teams seeking a scalable way to evaluate bedside technologies with intended users.

• Adapts traditional laboratory-based usability testing into a portable, conference-based method.

• Combines standardized clinical tasks with multimodal measures of visual attention, workload, usability, and performance.

• Provides a reproducible framework for evaluating medical-device usability with frontline clinicians.

## Graphical abstract

**Figure:**
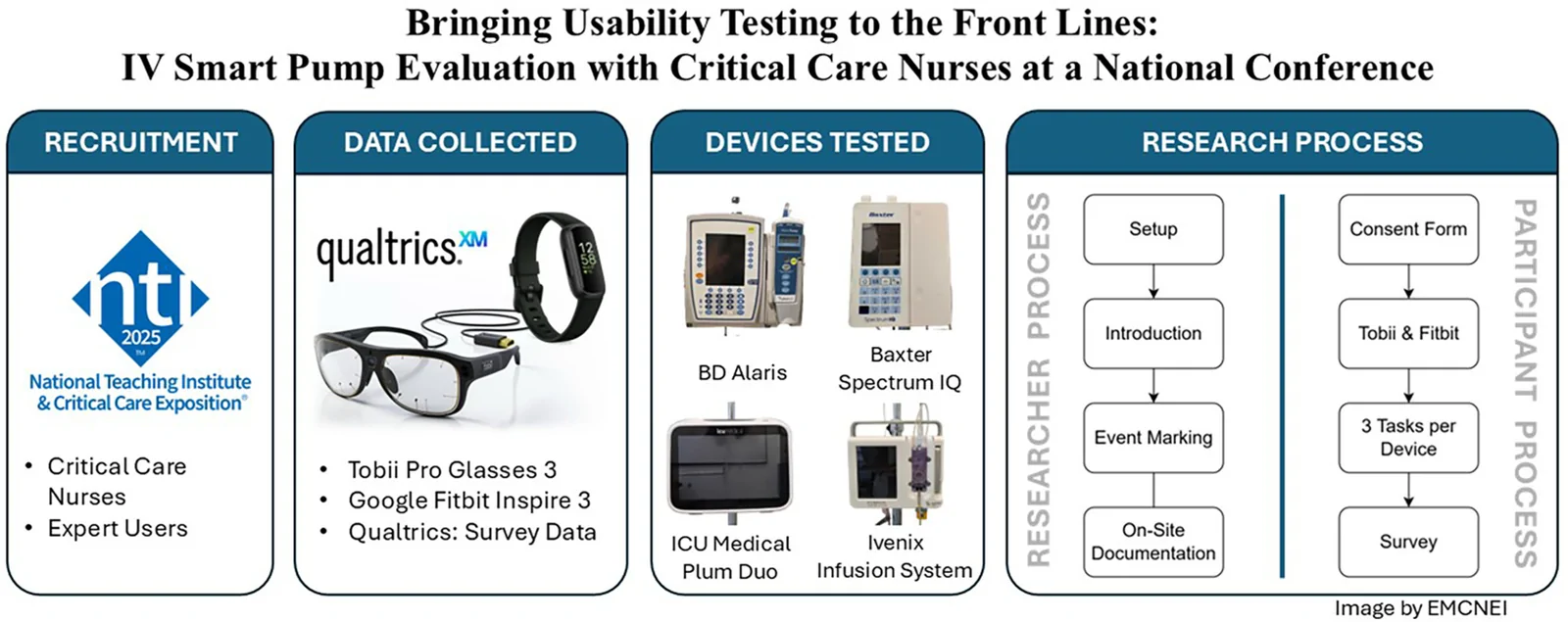

**Specifications table**

**Table:** 

**Subject area**	Medicine and Dentistry
**More specific subject area**	Human factors
**Name of your method**	Conference-based field-deployable usability testing method for intravenous smart pump evaluation
**Name and reference of original method**	Not applicable
**Resource availability**	Not applicable

## Background

Improving Intravenous smart pump (IVSP) usability is a fundamentally important patient-safety priority because usability challenges such as programming accuracy, clarity of interface cues, and alarm behavior directly influence nurses’ ability to deliver safe and timely infusions. Instead of treating usability as a technical feature, human-factors research considers it as a core component of medication-safety performance, where IVSPs that require excessive navigation or produce unclear feedback increase the likelihood of workflow disruptions and unnecessary alarms and alerts at the bedside [[1], [2], [3], [4]]. National guidance further elevates the role of usability in infusion safety. The U.S. Food and Drug Administration human factors engineering (HFE) guidance specifies that medical devices should be evaluated with representative users performing realistic tasks in intended use environments to ensure that interface design supports safe and effective operation [5]. Similarly, the Institute for Safe Medication Practices Smart Pump Guidelines emphasize designing IVSP workflows that minimize nuisance alerts, support accurate programming, and reduce reliance on workarounds [6]. The Infusion Nurses Society Standards further underscore that device selection and configuration should align with human factors principles to promote safe, uninterrupted infusion therapy [7].

However, there are limited examples in the literature of practical, scalable methods for conducting these kinds of usability evaluations with critical care nurses. To address this gap, the Elaine Marieb Center for Nursing and Engineering Innovation (EMCNEI) developed a field-deployable usability testing framework that brought a controlled simulation environment to the 2025 American Association of Critical Care Nurses National Teaching Institute in New Orleans, LA. This cost-effective approach enabled rigorous, standardized evaluation while reaching a broader and more diverse sample of experienced critical care nurses.

As the primary end-users of IVSPs in high-acuity settings, critical-care nurses routinely manage complex infusions, titrations, alarms, and competing clinical demands that shape how IVSPs are used in practice. Recent IVSP usability work has found that critical-care nurses’ interaction patterns, error-prevention strategies, and expectations for interface design are shaped by their high-acuity roles, making them essential participants in device evaluation and procurement decisions [8,9].

A conference-based recruitment approach extends these principles by enabling participation from critical care nurses across multiple states, hospital systems, and practice environments, thereby enhancing the ecological validity and generalizability of usability findings. In contrast to traditional simulation-center studies that typically recruit from a single organization or limited geographical region, this model preserves experimental control while engaging a broader and more representative segment of the critical care nursing workforce.

The purpose of this methods paper is to describe the design, implementation, and feasibility of a conference-based IVSP usability testing framework, and to present a replicable model for researchers, manufacturers, and health systems seeking to strengthen the evaluation of medical device usability for clinical use. The usability framework presented in this paper focuses on task performance as a core dimension of usability and incorporates additional measurements, including subjective workload, self-reported healthcare system usability scores, and physiological monitoring, to provide more insight into the factors that influence IVSP usability.

## Method details

### Sample and setting

A national sample of experienced (minimum of 2 years of experience using IVSP) critical-care nurses attending the American Association for Critical-Care Nurses (AACN) 2025 National Teaching Institute & Critical Care Exposition (NTI) conference, currently practicing in adult critical care, were recruited for participation in the study. Only users of either the BD Alaris or the Baxter Spectrum IVSP were recruited, so that each critical care nurse participant was familiar with only one of the four IVSPs used in the study. There was no attempt to control or recruit for age range, gender, ethnicity, or any other participant demographics.

Testing occurred over a period of four days. Pre-conference outreach via email using existing professional critical-care nurse contacts and social media was used to schedule participants for the first day of testing. On-site recruitment at the conference was used to schedule participants for the remaining three testing days.

Recruitment at a national critical-care nursing conference allowed access to a geographically and institutionally diverse group of ICU nurses from multiple states and health systems, enhancing the ecological relevance of the usability findings.

### Study design

This study used a within-subjects experimental design, in which every participant completed the full set of programming tasks on all four IVSPs. A within-subjects structure was selected to allow direct comparison of workload, usability, and programming performance across devices while controlling for individual differences in clinical experience and pump familiarity. Participants were not coached, corrected, or trained on any specific pump during task performance. This approach was intended to preserve natural interaction patterns and to evaluate the intuitiveness of each IVSP user interface.

## Equipment and materials

### Intravenous smart pumps

**Fig. 1:**
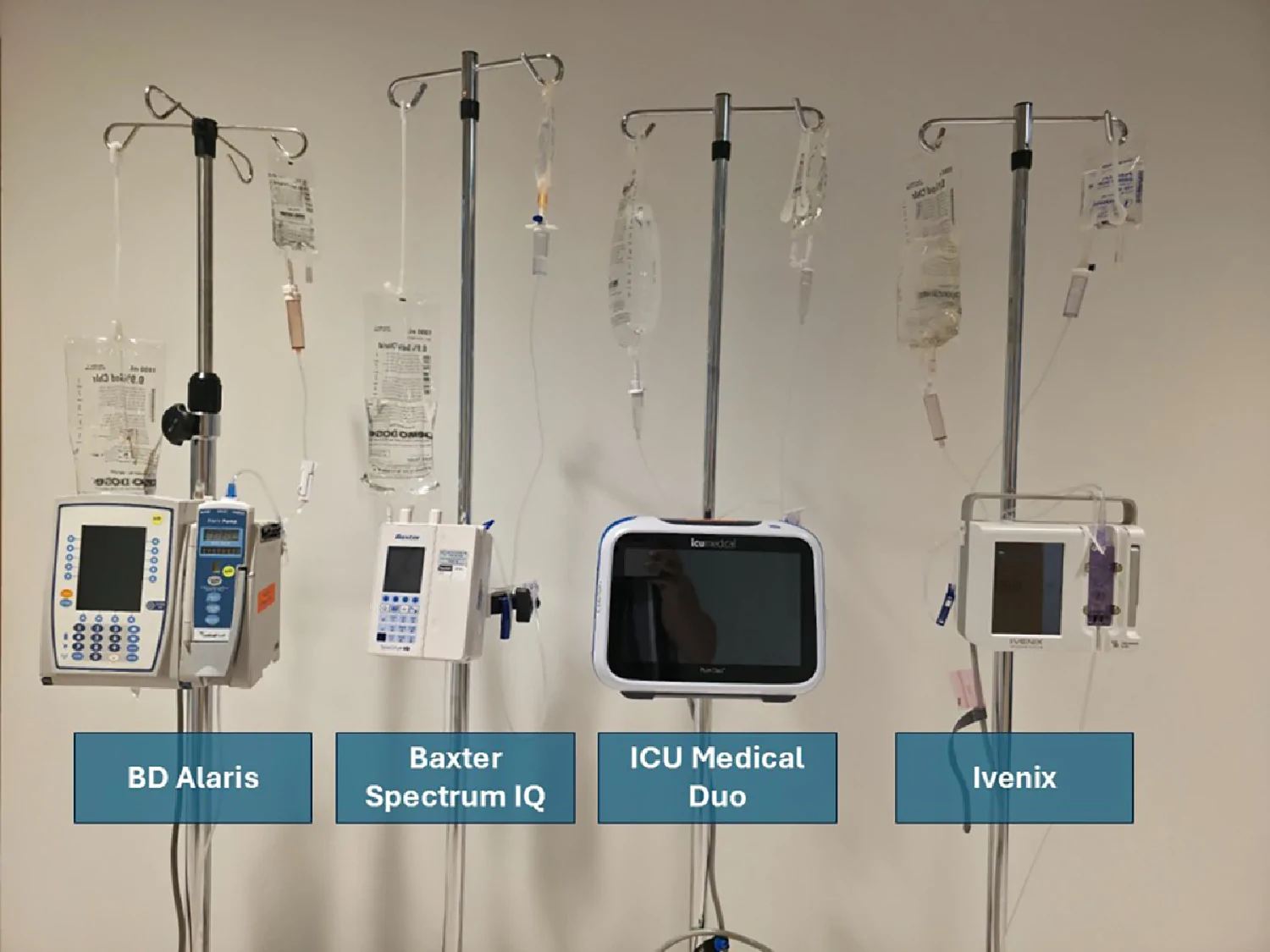
IV smart pumps in order of appearance BD Alaris (left), Baxter Sigma Spectrum IQ, ICU Medical Duo, Ivenix Infusion System (right) (photo used with permission from the Elaine Marieb Center for Nursing and Engineering Innovation, University of Massachusetts Amherst).

Four commercially available large-volume IVSPs were included: Baxter Spectrum IQ, BD Alaris, ICU Medical DUO, and the Ivenix Infusion System (Fig. 1). All IVSPs were set up with standardized demo drug libraries and configurations appropriate for simulated ICU infusions.

The Baxter Spectrum IQ and BD Alaris IVSP were selected because they represent approximately 80–85% of IVSPs currently used in US acute care. Although these two IVSPs are widely adopted, they use linear peristaltic IVSP technology, which is rooted in legacy design with limitations that have been found to constrain usability and safety in complex clinical environments [[10], [11], [12], [13], [14]].

The ICU Medical DUO and the Ivenix Infusion System are the two newest FDA-approved IVSP systems. These two cassette-based IVSPs offer advances in usability through simplified setup, more intuitive interfaces, integrated safety features, and touchscreen operation, and were both developed using the latest FDA human factors guidance. They were specifically designed to reduce programming complexity and support safer, more efficient infusion workflows [14].

### Eye tracking

**Fig. 2:**
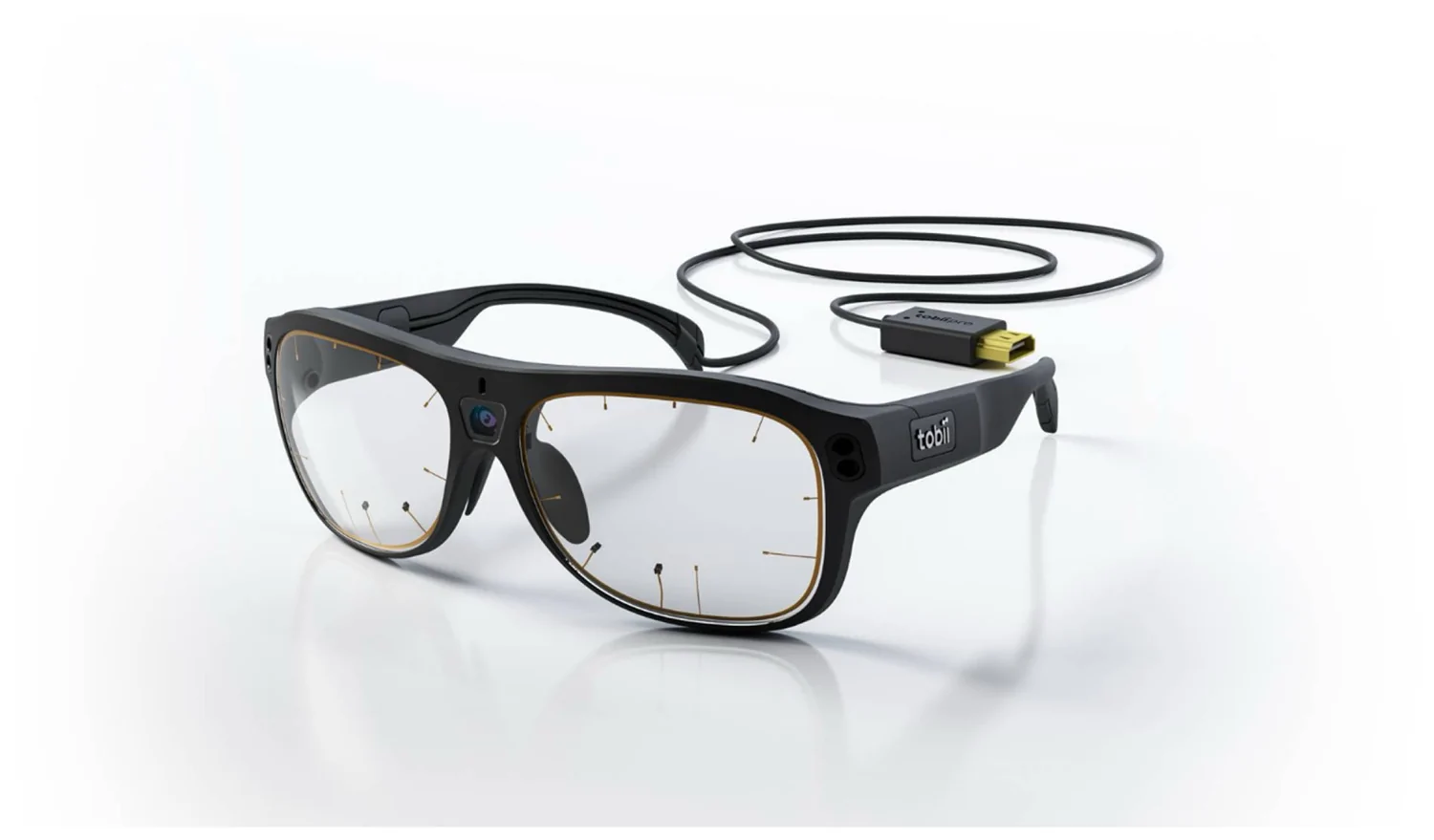
Tobii pro glasses 3 wearable eye‑tracking device.

In this study, objective measures of visual attention were captured using Tobii Pro 3 wearable eye-tracking glasses (Tobii Pro 3, Stockholm, Sweden) (Fig. 2). The eye-tracking system consists of wearable glasses, a recording unit, controller software, and analyzer software. The head unit uses a high-definition scene camera, a microphone, eye- tracking sensors, and IR illuminators. Tobii Glasses Controller Software was used for calibration, recording, and video export. Pro Lab Analyzer Edition (version 1.217) software was used for post-data collection analysis. Eye tracking is a non-invasive technique where an individual’s eye movements are measured and analyzed to provide objectively measured information on visual attention and gaze fixation during task performance [15]. Eye tracking is becoming a more frequently used technology in usability research to better understand how people process and understand visual and display-based information, offering objective insight into visual attention during device interaction [16,17]. The eye-tracking device provides a multitude of biometric data related to the eye. Not only does it capture a first-person view of the participant and their gaze heatmap, but the eye-tracking system can provide information such as saccade rate, blink rate, and fixation duration, which are crucial in understanding the workload during a task [18]. Eye tracking with the Tobii Pro Glasses 3 has demonstrated strong performance across both controlled and dynamic environments. Although the manufacturer does not publish detailed drift or slippage metrics, independent evaluations concluded that the system has acceptable accuracy [19]. In clinical laboratory settings, prior work showed that the device produces mean vergence estimates within approximately 1° of true vergence demand across nearly all tested angles, with deviations occurring only at the most extreme vergence positions. Additional studies reported that the glasses maintain high accuracy during dynamic motion, including walking and free head movement, outperforming earlier wearable eye-tracking models [20]. Furthermore, Pettenella et al. demonstrated that Tobii Pro Glasses 3 are least affected by slippage across a range of induced motions, indicating that the device showed minimal drift even under movement-heavy conditions [21]. Data from eye-tracking measurements can assist usability researchers in user-interface design to improve ease of use and reduce errors at the human-device interface. Furthermore, its use in IVSP usability research is novel. During our study, Tobii research specialists provided their expertise on site for guidance on system use during the study, calibration procedures, and the data collection process to minimize measurement error and optimize data quality in this field-based setting.

### Physiologic monitoring

Physiologic data were collected using a Fitbit Inspire 3 (San Francisco, CA) wrist-worn device placed on the participant’s non-dominant wrist during all study procedures. The device continuously recorded heart rate (HR) during baseline and task-performance periods. Heart rate data were exported after data collection and used to derive heart rate variability (HRV)-related measures during subsequent analysis. Heart rate monitoring was included as a supplementary objective indicator of physiologic response and cognitive workload during IVSP interaction.

Because Fitbit collects biometric data, it requires academic or non-profit researchers to apply for a health research application to access extensive Fitbit data via the Web API (Application Programming Interface). The research application was approved by the Clinical Research Operations Program Manager at Google. A server-side application was registered, and data was collected via the API and Google Takeout. A Python script was developed to retrieve heart rate data from the Fitbit API and merge it with Google Takeout exports using each sample’s timestamp as the alignment key. These datasets, together with the eye-tracking output extracted from Tobii Pro Lab, were then validated and pre-processed using the custom proofing system available on GitHub [22]. The tool enables systematic review of eye-tracking event markers, identifying duplicated task labels (e.g., *Task 1, Task 2*) and verifying their temporal order (*Start* comes before *End*), which defines the duration of each task segment. The program also synchronizes heart rate data with the eye-tracking timestamp. Because Fitbit heart rate measurements are not uniformly sampled and are partially obscured by proprietary preprocessing, the resulting data average only 0.2 Hz, substantially lower than Tobii’s 100 Hz sampling rate. Using synchronized event markers from the eye-tracking dataset, a visual inspection was performed to confirm the logical alignment between heart rate changes and task events.

### Subjective workload

The National Aeronautics and Space Administration Task Load Index (NASA-TLX) is the most widely used multidimensional subjective workload instrument. Early work and subsequent reviews have shown acceptable internal consistency (Cronbach’s α typically ≥ 0.70) and good sensitivity to experimentally important differences in workload across tasks and systems [[23], [24], [25], [26]]. The NASA-TLX evaluates workload across six dimensions: Mental Demand, Physical Demand, Temporal Demand, Performance, Effort, and Frustration (Table 1). In the original instrument, NASA-TLX was scored using a two-step process involving both weighting and rating. However, a modified and validated version of the NASA-TLX, using only the ratings (raw NASA-TLX), has become much more commonly used because it is more time-efficient and simpler to apply [27].

**Table 1:** Modified raw NASA-TLX questions and rating scale.

Subscale Title	Modified Definition	Subscore
Mental Demand	How mentally demanding was the task?	Low (0) - High (100)
Physical Demand	How physically demanding was the task?	Low (0) - High (100)
Temporal Demand	How hurried or rushed was the pace of the task?	Low (0) - High (100)
Performance	How successful were you in accomplishing what you were asked to do?	Good (0) - Poor (100)
Effort	How hard did you have to work to accomplish your level of performance?	Low (0) - High (100)
Frustration	How insecure, discouraged, irritated, stressed, and/or annoyed were you during the task?	Low (0) - High (100)

In this study, participants completed the raw NASA-TLX after completing the standardized programming tasks on each IVSP. Each subscale was rated on a 0–20 scale. Total workload for each device was calculated by summing the ratings of the subscales, yielding a total workload score ranging from 0 to 100, with higher scores indicating greater perceived workload.

### IVSP system usability

IVSP system usability was measured by self-report using the Healthcare Systems Usability Scale (HSUS), a 22-item questionnaire developed to evaluate the usability of clinical decision support systems (CDSS) and health information systems [28]. For this study, we administered two of the four HSUS subscales: Workflow integration and User control because those domains most directly aligned with our focus on bedside IVSP usability.

Each HSUS item was rated on a 5-point Likert scale ranging from 1 (strongly disagree) to 5 (strongly agree), with higher scores indicating better perceived usability. Responses were summed according to the instrument scoring guidelines to calculate the Workflow Integration (6 items; score range 6–30) and User Control (4 items; score range 4–20) subscale scores for each IVSP. Mean subscale scores were then calculated across participants for each device, with higher scores indicating better perceived usability.

## Usability testing environment and study workflow

**Fig. 3:**
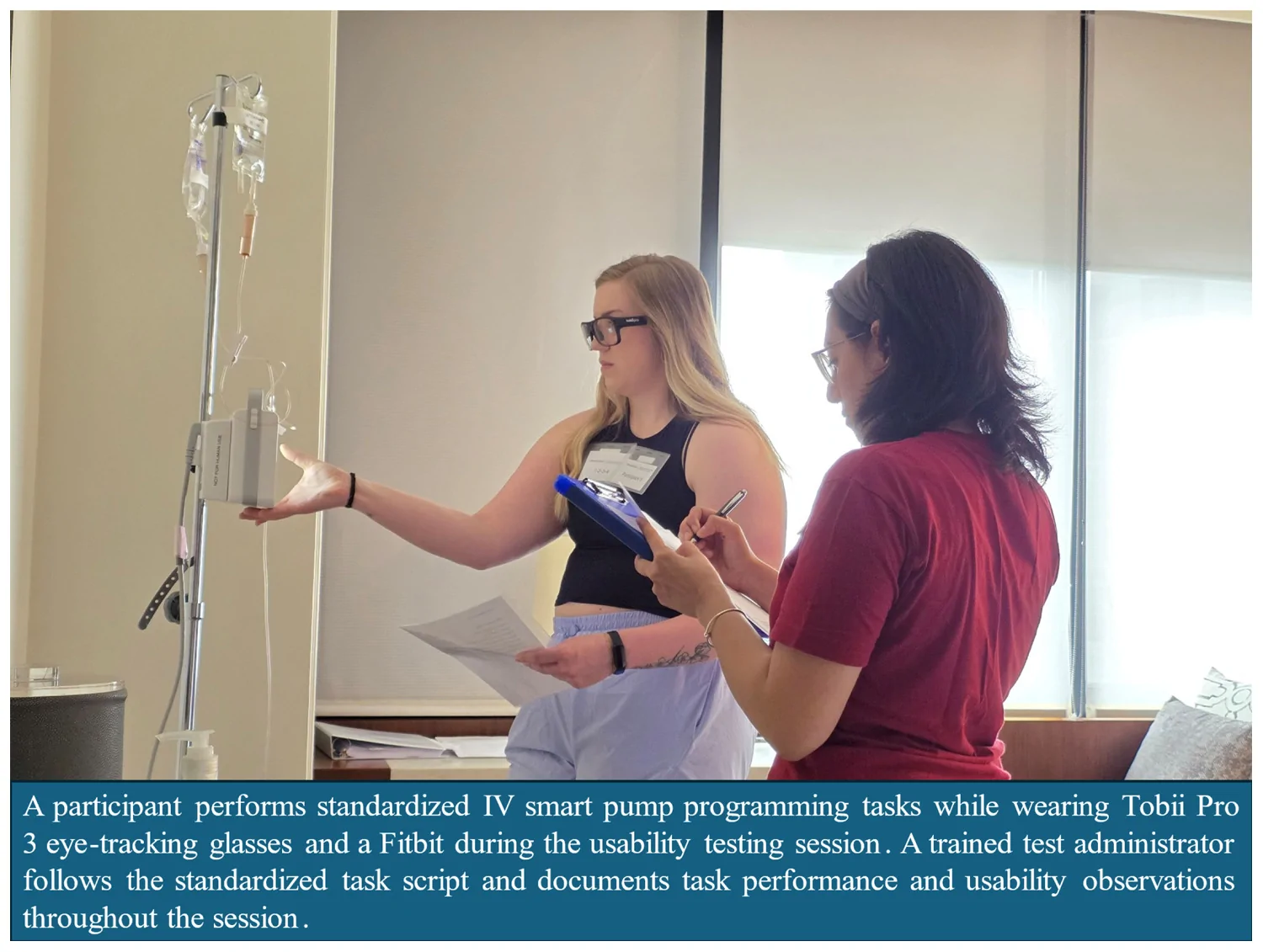
Usability testing station and task administration. (photo used with permission from the Elaine Marieb Center for Nursing and Engineering Innovation, University of Massachusetts Amherst).

A total of four usability testing stations were used. The stations contained a single IVSP, an IV pole, standardized IV tubing, infusion bags, and scripted task materials. Each room was staffed by trained members of the research team responsible for test administration, timing, and observational data collection (Fig. 3). To ensure consistency across devices and participants, all pumps were configured using the same physical layout and environmental conditions. Pumps were clamped to IV poles positioned 53 inches from the floor. In compliance with the manufacturer’s IVSP system setup requirements, primary infusion bags were hung 20–22 inches above the pump with labels facing the participant, while secondary infusion bags were connected to the primary tubing and positioned at the same height. For the Baxter Spectrum IQ and BD Alaris pumps, a secondary hanger was attached to the IV pole in the folded position for use during secondary infusion tasks.

The testing rooms were arranged to minimize environmental variability that could interfere with usability assessment or eye-tracking performance. Upon arrival, participants were greeted by a member of the research team, screened for eligibility, and provided with a study overview before written informed consent was obtained. Participants were assigned identification numbers and randomly selected one of four predetermined pump-order sequences by drawing a printed sequence slip from a basket. The selected sequence determined the order in which the participant completed the programming tasks on the four IVSPs. The four predetermined sequences varied the testing order across participants to minimize potential bias related to the order of device evaluation. The participants were then fitted with Tobii Pro 3 eye-tracking glasses and a Fitbit Inspire 3 wrist device on the non-dominant wrist. Eye-tracking calibration was performed according to the manufacturer's procedures before testing began. Participants who wore corrective lenses were asked to provide their current prescription information to support appropriate lens insert selection and calibration adjustments by the Tobii technical team. A brief baseline recording period was conducted prior to task initiation, with participants standing quietly. During this period, heart rate data were collected for subsequent comparison with task-related physiologic responses.

After calibration, Participants then progressed through the four IVSP testing stations in their assigned order, accompanied by an eye-tracking specialist who monitored signal quality and calibration stability. At each station, participants completed three standardized IVSP programming tasks representing increasing levels of cognitive and operational complexity. Consistent with best practices for usability testing, test administrators used a standardized test administrator script to ensure consistent task instructions, prompts, timing procedures, and error documentation across pumps and participants.

Using the Tobii Recording Software, event markers were added to denote significant events that occurred during the experiment. The event markers were placed for application and removal of the Fitbit, IVSP programming start and end, and task start and end. The marker names were set consistently across all eye-tracking recording devices used during the experiment. This enabled inter-device uniformity, which was used as a synchronization point for both eye-tracking devices and Fitbit recordings during data analysis. These events were marked by the corresponding researcher who maintained the eye-tracking device during the experiment.

Participants were instructed to verbalize their thought processes during interaction with the pumps using a think-aloud approach. Research staff documented participants' verbalizations and notable comments in real time while simultaneously observing task performance. Audio recordings were not obtained. The documented think-aloud comments were used to provide contextual information for interpreting participant actions, workflow interruptions, and observed usability issues during the usability evaluation and were not subjected to formal qualitative coding or thematic analysis. Eye tracking and heart rate data were recorded continuously throughout task performance, while research staff documented task completion time, programming errors, workflow interruptions, and notable usability observations. Task timing began when the participant first interacted with the pump to initiate the assigned programming task and ended when the infusion was successfully initiated or when the predetermined 5 min time limit was reached.

Observational outcomes were defined before data collection using the standardized Test Administrator Guide. A programming error was defined as an incorrect programming action or omission that resulted in an incorrect programming sequence, required correction, or prevented successful completion of the assigned task. A workflow interruption was defined as any event that disrupted the normal progression of the programming task, including device alarms or procedural deviations. Task completion was defined as successful programming of the assigned infusion according to the specified clinical scenario and initiation of the infusion. Task failure was defined as failure to successfully complete and initiate the assigned programming task within the allotted 5 min time limit. Notable usability observations included participant behaviors or verbalizations documented by the test administrator that reflected navigation difficulties, hesitation, confusion, or other interface-related usability issues observed during the participant think-aloud. After completing all testing stations, participants completed the electronic surveys and received a $100 electronic honorarium.

The three standardized programming tasks were designed to represent progressively increasing levels of clinical complexity while reflecting common IV medication administration workflows encountered by critical care nurses. The clinical scenarios, programming requirements, expected task endpoints, and rationale for each task are summarized in Table 2.

**Table 2:** Clinical scenarios and programming tasks included in the standardized IVSP usability protocol.

Task	Clinical Scenario	Medication / Fluid	Programming Requirements	Expected Endpoint	Complexity Rationale
Primary infusion	Newly admitted patient requiring maintenance IV fluids	IV Plain (Normal saline)	Turn on the pump, access the medical-surgical drug library, program a primary infusion at 125 mL/hr with a VTBI of 250 mL, and initiate the infusion.	Primary infusion initiated correctly using the appropriate drug library and programmed infusion parameters.	Represents a routine, low-complexity task performed frequently in clinical practice and evaluates basic navigation, drug library access, and infusion programming.
Secondary infusion	Patient with bilateral pneumonia requiring immediate IV antibiotics	Cefazolin 1 g/100 mL	Program a secondary infusion over 30 min while maintaining the primary infusion. Device-specific secondary infusion workflow (e.g., bag positioning and clamp management for linear pumps) was completed according to the manufacturer's operating requirements.	Secondary infusion initiated with correct medication, infusion duration, and appropriate secondary infusion setup.	Represents an intermediate-complexity task requiring management of concurrent infusions and device-specific workflow steps that are common sources of programming errors.
Insulin titration	Critically ill patient with persistent hyperglycemia requiring continuous insulin therapy	Regular insulin infusion	Stop the existing infusions, switch to the critical care drug library, initiate an insulin infusion at 4 U/hr, then sequentially titrate to 6 U/hr and 8 U/hr in response to simulated blood glucose changes.	Weight-based insulin infusion initiated and successfully titrated according to the changing clinical scenario.	Represents the highest-complexity task, requiring drug library changes, repeated programming decisions, and dose titration.

IV: Intravenous; VTBI: Volume to be infused; U/hr: Units per hour.

## Methodological considerations

Several potential sources of methodological error were anticipated, and corresponding strategies were built into the protocol to mitigate them. Although testing occurred in private rooms, environmental noise and lighting (e.g., hallway noise, variable ambient light, glare on pump screens) could distract participants or affect eye-tracking quality. Participant fatigue was also possible given that each nurse completed three tasks on four pumps during a single session, and conference-related time pressure could influence pace and engagement.

To address these issues, the study employed several mitigation strategies. Testing rooms were arranged to minimize visual clutter and background noise, with doors closed and lighting adjusted to reduce glare and support stable eye tracking. Eye-tracking calibration was performed at the start of each session and rechecked whenever glasses were adjusted or data quality suggested drift, with brief pauses used to recalibrate as needed. All task instructions and test administrators used a standardized script. Participants were given short pauses between IVSP test stations to limit fatigue accumulation.

## Data handling

All data streams, including eye-tracking files, task-performance logs, survey responses, and physiologic recordings, were labeled with unique study codes and stored on encrypted, password-protected servers at the University of Massachusetts Amherst. No direct identifiers were contained in the analytic datasets. Raw gaze data were processed using Tobii Pro software and exported as fixation- and event-level files for statistical analysis.

Data quality checks were conducted immediately after collection and again prior to analysis. Survey datasets were reviewed for completeness and logical consistency. All enrolled participants completed the full protocol, and there were no missing survey or task-performance data.

Because each participant completed the standardized programming tasks on all four IVSPs, the study employed a repeated-measures (within-subjects) design. The methodology generated multiple complementary datasets, including questionnaire responses, eye-tracking metrics, physiologic measurements, and programming performance outcomes. Each dataset was analyzed using analytical methods appropriate for the corresponding outcome measures while accounting for the repeated observations obtained from each participant. Detailed statistical analyses for each outcome are reported in the corresponding manuscripts derived from this protocol.

For eye-tracking data, although the sample-based accuracy and precision are not available, a post-study analysis using Tobii Pro Lab™ quantifies the proportion of valid gaze samples relative to tracking dropouts. In this study, only periods with reliable, computable gaze data were used for subsequent analysis, ensuring that all reported results reflect stable and high-quality eye-tracking performance.

## Lessons learned

Implementation of this conference-based usability testing framework yielded several important practical and methodological insights. First, national conference settings can serve as highly effective venues for recruiting a diverse sample of experienced clinicians. Access to critical care nurses from multiple states, hospital systems, and practice environments enhanced the breadth of perspectives captured and the generalizability of the findings. The structured scheduling approach enabled efficient, high-throughput data collection while supporting protocol consistency.

Careful attention to physical setup and environmental control is essential when translating simulation-based usability testing into a field setting. Standardizing IVSP positioning, setup, lighting, and room configuration was critical to ensuring comparability across participants and devices, and to maintaining eye-tracking data and measurement fidelity and quality.

Integrating wearable technologies such as eye tracking and physiologic monitoring into a mobile testing environment is feasible. However, it does require dedicated technical expertise and real-time oversight. Collaboration with Tobii was instrumental in optimizing calibration procedures, minimizing data loss, and troubleshooting issues such as drift or fit. Assigning trained personnel to monitor data quality throughout each session was important for maintaining usable datasets.

Participant burden and fatigue need to be actively managed in within-subjects designs conducted outside of traditional laboratory settings. Scheduling buffer time between sessions, allowing brief rest periods between devices, and maintaining a predictable testing flow helped sustain engagement and performance across the full protocol. The overall session length was acceptable to participants while being respectful within the constraints of conference schedules.

The decision not to provide training or feedback allowed for a less biased assessment of interface intuitiveness. Framing the evaluation as a test of the device, not the individual, was important for reducing performance anxiety and positioning the participants as experts with the knowledge needed for IVSP testing. This encouraged more natural interaction patterns and think-aloud verbalizations.

Finally, multimodal data integration, combining task performance, eye tracking, physiologic measures, and self-reported workload, provided a richer and more comprehensive understanding of IVSP usability than any single method alone. While this approach introduced some complexity into data synchronization, management, and analysis, it also provided a novel and comprehensive approach to the study and comparison of the IVSP user interfaces. Together, these lessons demonstrate that conference-based usability testing is a viable and scalable approach for evaluating medical devices with frontline clinicians.

## Method validation

The method was implemented as described in the Method details section, demonstrating the feasibility of a field-deployable approach to usability testing with frontline clinicians. The procedural detail provided is intended to support adaptation and replication in future evaluations of bedside technologies.

## Limitations

Despite its strengths as a novel, field-deployable usability testing approach, this study has limitations that should be considered in the context of this approach. While testing was conducted in a controlled environment, it was a simulated setting where it is not possible to fully replicate the complexity of real-world bedside care. Participants were not exposed to common clinical interruptions, multitasking demands, time pressure associated with patient acuity, or team-based communication, all of which may influence IVSP interaction and cognitive workload in practice. As a result, observed usability patterns may differ from those occurring in fully dynamic clinical environments.

The conference-based recruitment model, while enabling access to a geographically and institutionally diverse sample of experienced critical care nurses, may introduce selection bias. Participants were self-selected conference attendees who may differ from the broader critical care nursing population with regard to engagement, expertise, or interest in professional development and technology. Additionally, time constraints associated with conference participation may have limited participant recruitment.

The within-subjects design required participants to complete multiple tasks across four different IVSPs within a single session. Although counterbalancing and scheduled breaks were used to mitigate order effects and fatigue, cumulative cognitive load and learning may have influenced performance, workload ratings, and interaction patterns across IVSP devices.

While eye tracking provided valuable objective data on visual attention and interface interaction, its limitations in using pupillometry measures are pertinent in this study. Pupillometry measures changes in pupil size over time, providing a non-invasive window into cognitive and physiological processes. Because the pupil dilates and constricts in response to mental effort, emotional arousal, and autonomic nervous system activity, pupillometry is used as an index of cognitive load, attention, fatigue, and stress. It has broad applications in research areas ranging from human factors and ergonomics to clinical neuropsychology.

An important limitation of pupillometry is its sensitivity to ambient light. The pupillary light reflex is the automatic constriction of the pupil in response to increased light. Because this reflex operates independently of cognitive or emotional state, fluctuations in ambient lighting can introduce error into pupillometric data, obscuring the smaller, task-related pupil changes that eye tracking is able to capture. Even subtle shifts in screen brightness, overhead lighting, or a participant's gaze direction toward a light source can produce pupil responses that are difficult to distinguish from those driven by cognitive processes. For this reason, controlled and consistent lighting conditions are considered essential for valid pupillometry measurement. Since this field deployment did not allow for effective control of ambient light, especially the backlighting from the IVSP devices, we chose not to use pupillometry data as a measure of stress or cognitive load.

The physiologic measure, heart rate, was collected using a consumer-grade wearable device. While this approach supported feasibility and participant comfort in a field setting, its precision is lower than that of clinical-grade monitoring systems. This is important when collecting raw data. The Fitbit does not allow access to raw heart rate data due to its proprietary signal processing algorithm. Therefore, even with a web API, only rough processed data is available for researchers. Although various studies [29] have concluded that the Fitbit is a viable heart rate monitoring solution, because the data is sparse(0.2 Hz) and processed, small changes in heart rate cannot be extracted, but macroscopic trends of heart rate can be used. Furthermore, since the on-board data is not accessible, a data sync to a mobile device is necessary after each experiment. Because there is no dedicated syncing button, the researcher has no choice but to rely on the device’s data syncing by refreshing the Fitbit app. Although processed data can be more useful for long-term measurements, a research-grade device for monitoring physiological measures would have provided higher precision for heart rate monitoring.

Finally, although standardized tasks were designed to reflect common IVSP infusion tasks, they cannot fully capture the variability and complexity of real-world medication administration.

## Ethics statements

This study involved human participants. All participants provided informed consent prior to participation. The protocol was reviewed and approved by the University of Massachusetts Amherst Institutional Review Board (IRB Protocol #:6389). Participation was voluntary, and all data were collected and stored in accordance with institutional and federal ethical guidelines.

## Acknowledgments

Funding for this study was provided by a faculty research grant from the University of Massachusetts Amherst and the Mary Logan Grant from the Association for the Advancement of Medical Instrumentation. The authors thank the EMCNEI for providing the photography used in the graphical abstract and study visuals.

## Declaration of generative AI and AI-assisted technologies in the manuscript preparation process

During the preparation of this work, the authors used Grammarly in order to improve grammar and clarity. After using this tool/service, the authors reviewed and edited the content as needed and take(s) full responsibility for the content of the published article.

## Declaration of competing interest

The authors declare the following financial interests/personal relationships which may be considered as potential competing interests:

Karen Giuliano has received a donated IV smart pump from ICU Medical for the IV smart pump lab at the Elaine Marieb Center for Nursing and Engineering Innovation. Karen Giuliano has also received travel support and payment for time to present research findings from ICU Medical. No other authors have any competing interests to declare.

## Data Availability

Data will be made available on request.
